# Supplementary material for: The protective effect of carbamazepine on acute lung injury induced by hemorrhagic shock and resuscitation in rats
Source: PLoS One. 2024 Oct 23;19(10):e0309622. doi: 10.1371/journal.pone.0309622 (PMC11498730; doi:10.1371/journal.pone.0309622)
Supplement: S2 File — This file depicts a timeline for the experimental procedure involving the administration of CBZ, CQ, and DMSO in this study. (PDF) [file pone.0309622.s002.pdf]

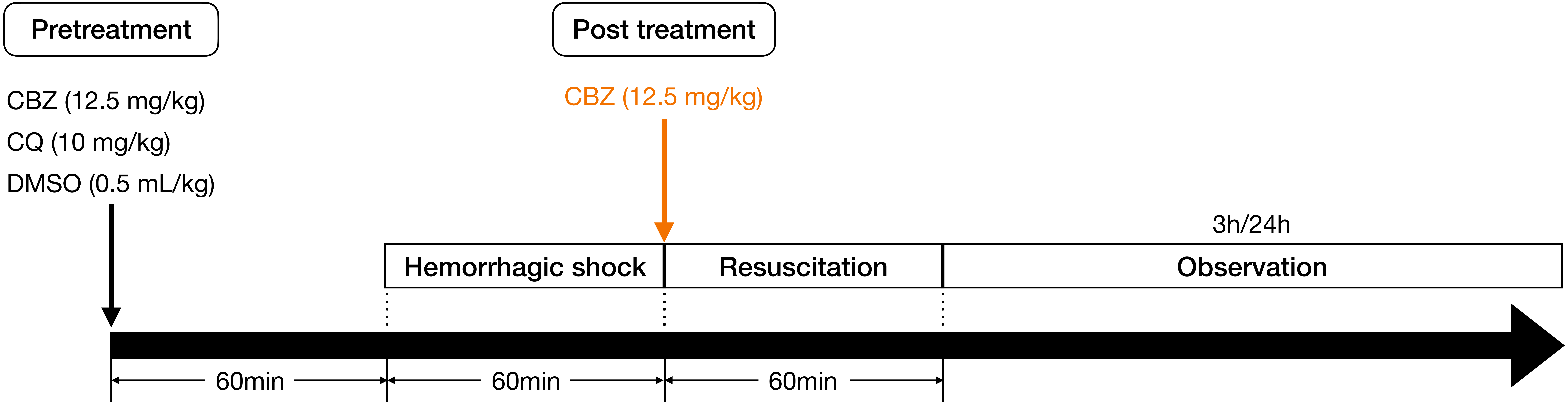

Pretreatment
CBZ (12.5 mg/kg)
CQ (10 mg/kg)
DMSO (0.5 mL/kg)
Post treatment
CBZ (12.5 mg/kg)
3h/24h
Hemorrhagic shock
Resuscitation
Observation
60min
60min
60min
