# Supplementary figures and images for: The protective effect of carbamazepine on acute lung injury induced by hemorrhagic shock and resuscitation in rats
Source: PLoS One. 2024 Oct 23;19(10):e0309622. doi: 10.1371/journal.pone.0309622 (PMC11498730; doi:10.1371/journal.pone.0309622)

A

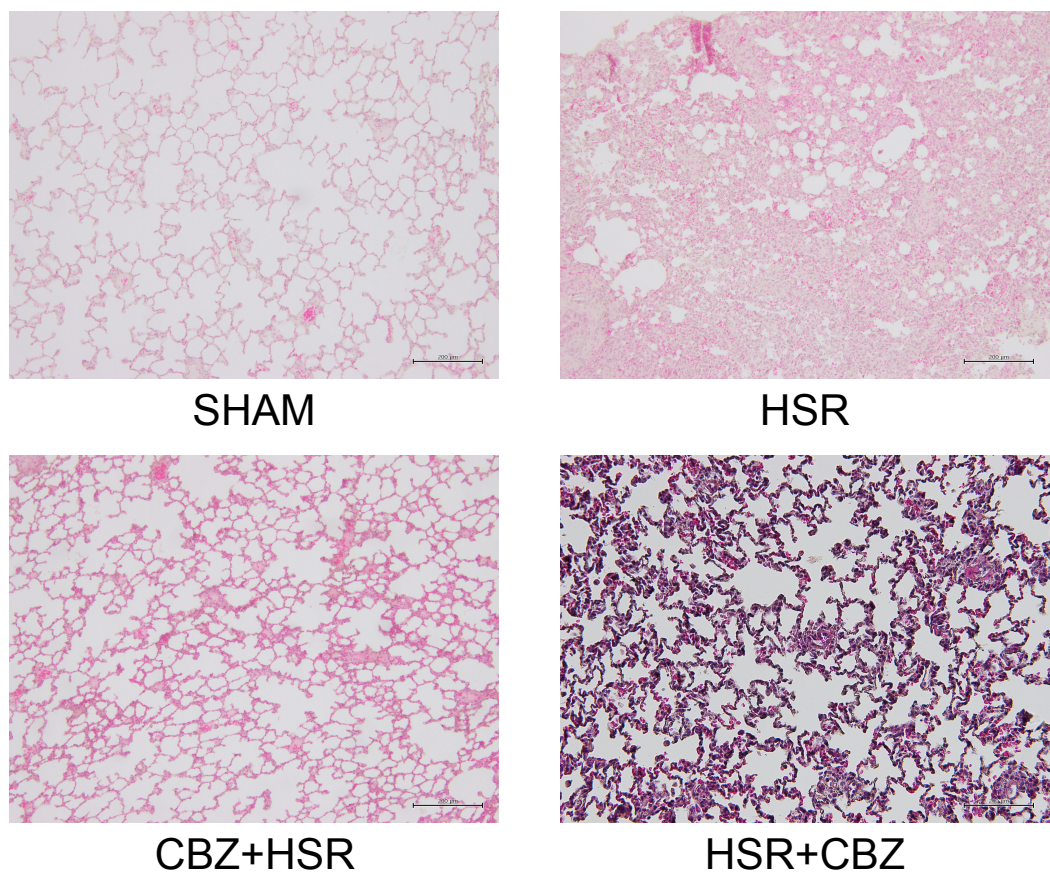

B

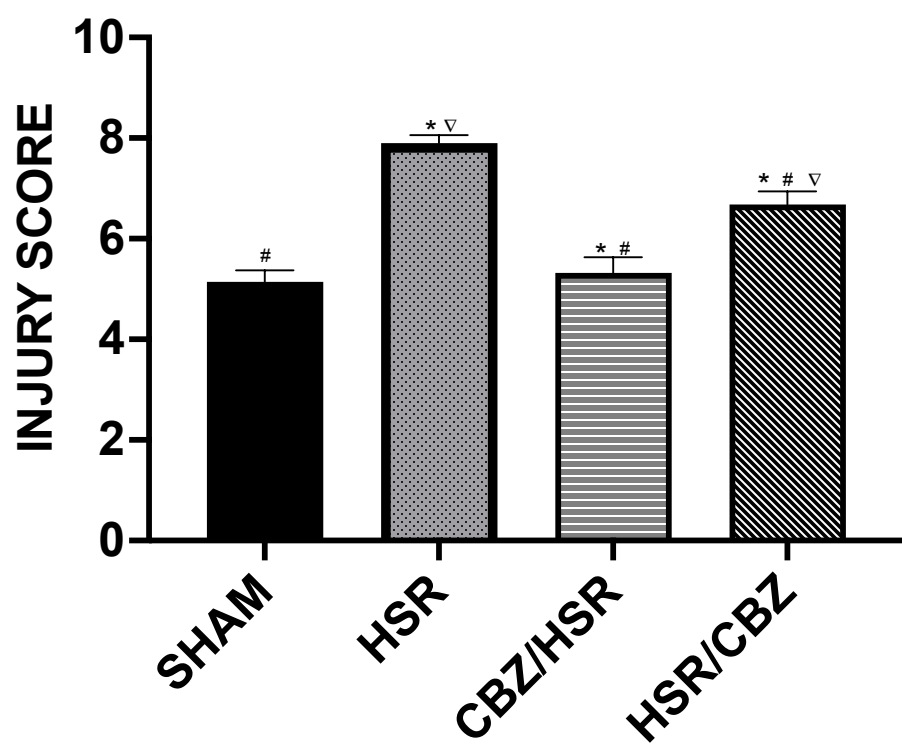

Supplement: S3 File — Histological examination of lung injury in four groups. Rats were sacrificed 24 h after HSR, and lung tissues were collected for histological analysis. Lung sections were observed with the microscope (original magnification × 200). (A) Histological alterations, including congestion, edema, inflammation, and hemorrhage, were noted in the HSR group. However, these histopathological changes were significantly ameliorated after treatment with CBZ, while the administration of CBZ after shock also improved the tissue damage compared with the HSR group. (B) By analyzing the changes in pathological sections of each group, lung injury score was calculated. Consistent with the histopathological results, the lung injury scores were notably elevated in the HSR group; however, administration of CBZ before shock and after shock both markedly decreased these scores. Data in each analysis are presented as the mean ± SEM (n = 5). *p < 0.05 vs SHAM, #p < 0.05 vs HSR and ∇p < 0.05 vs CBZ/HSR. CBZ, carbamazepine; HSR, hemorrhagic shock and resuscitation; HE, hematoxylin and eosin; SEM, standard error of the mean. (PDF) [file pone.0309622.s003.pdf]
