# Supplementary material for: The protective effect of carbamazepine on acute lung injury induced by hemorrhagic shock and resuscitation in rats
Source: PLoS One. 2024 Oct 23;19(10):e0309622. doi: 10.1371/journal.pone.0309622 (PMC11498730; doi:10.1371/journal.pone.0309622)
Supplement: S4 File — This file shows the original uncropped and unadjusted images underlying all blot reports. (PDF) [file pone.0309622.s004.pdf]

Tha samples were loaded in the order of their numbers.
In the figure 1-16 are shown.
Protein： LC3

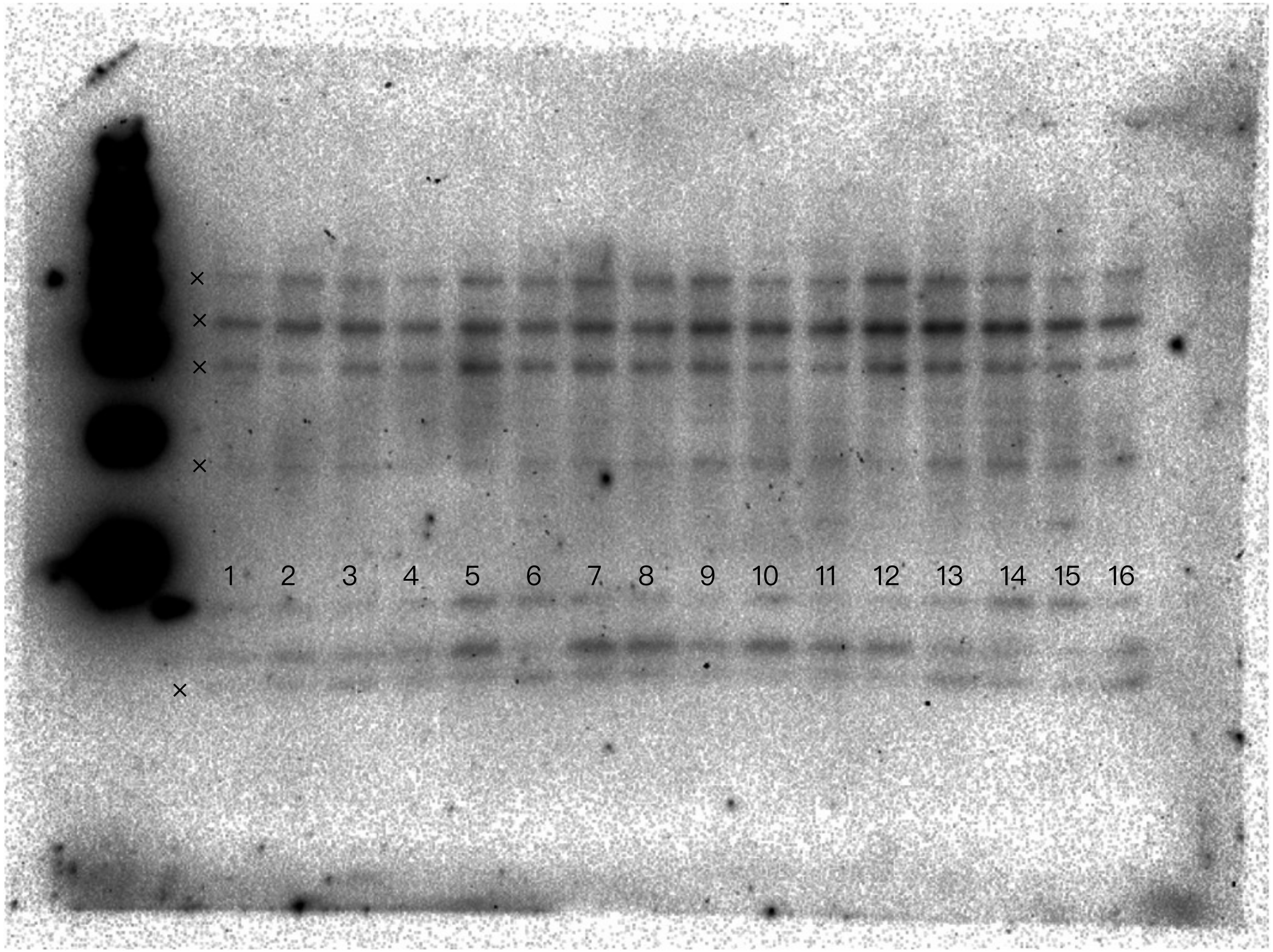

16KD
14KD

Sample ID
1 2019070401
2. 2019070501
3. 2019070502
4. 2019032202
5. 2018120701
6. 2018120702
7. 2018121701
8. 2018122701
9. 2019070801
10. 2019070901
11. 2019071001
12. 2019050402
13. 2019081101
14. 2019081201
15. 2019080201
16. 2019082801

Antigen-antibody complexes were stained with Clarity Western ECL substrate(Bio-Rad) and visualization was performed using an image scanner(ChemiDoc XRS Plus Imaging System, Bio-Rad)

Tha samples were loaded in the order of their numbers.
In the figure 1-16 are shown.
Protein： Beclin-1

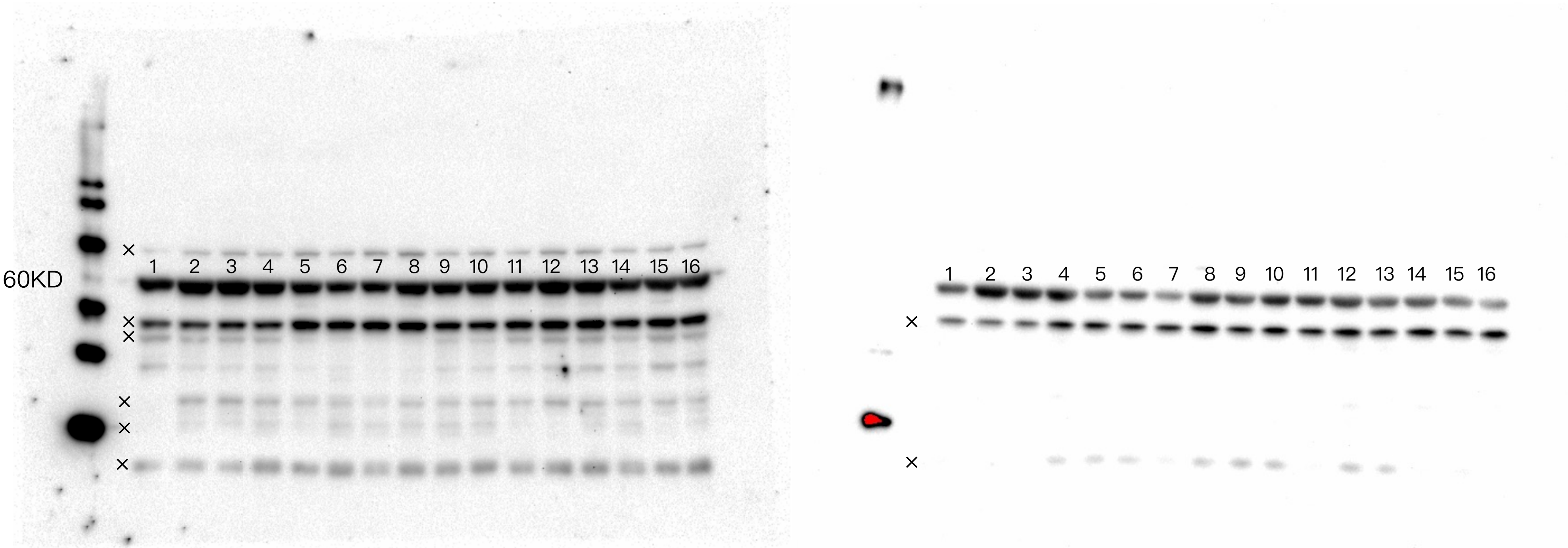

Antigen-antibody complexes were stained with Clarity Western ECL substrate(Bio-Rad) and visualization was performed using an image scanner(ChemiDoc XRS Plus Imaging System, Bio-Rad)

Sample ID

1. 2019070401
2. 2019070501
3. 2019070502
4. 2019032202
5. 2018120701
6. 2018120702
7. 2018121701
8. 2018122701
9. 2019070801
10. 2019070901
11. 2019071001
12. 2019050402
13. 2019081101
14. 2019082901
15. 2019081201
16. 2019080201

Tha samples were loaded in the order of their numbers.
In the figure 1-16 are shown.
Protein: Atg12-Atg5

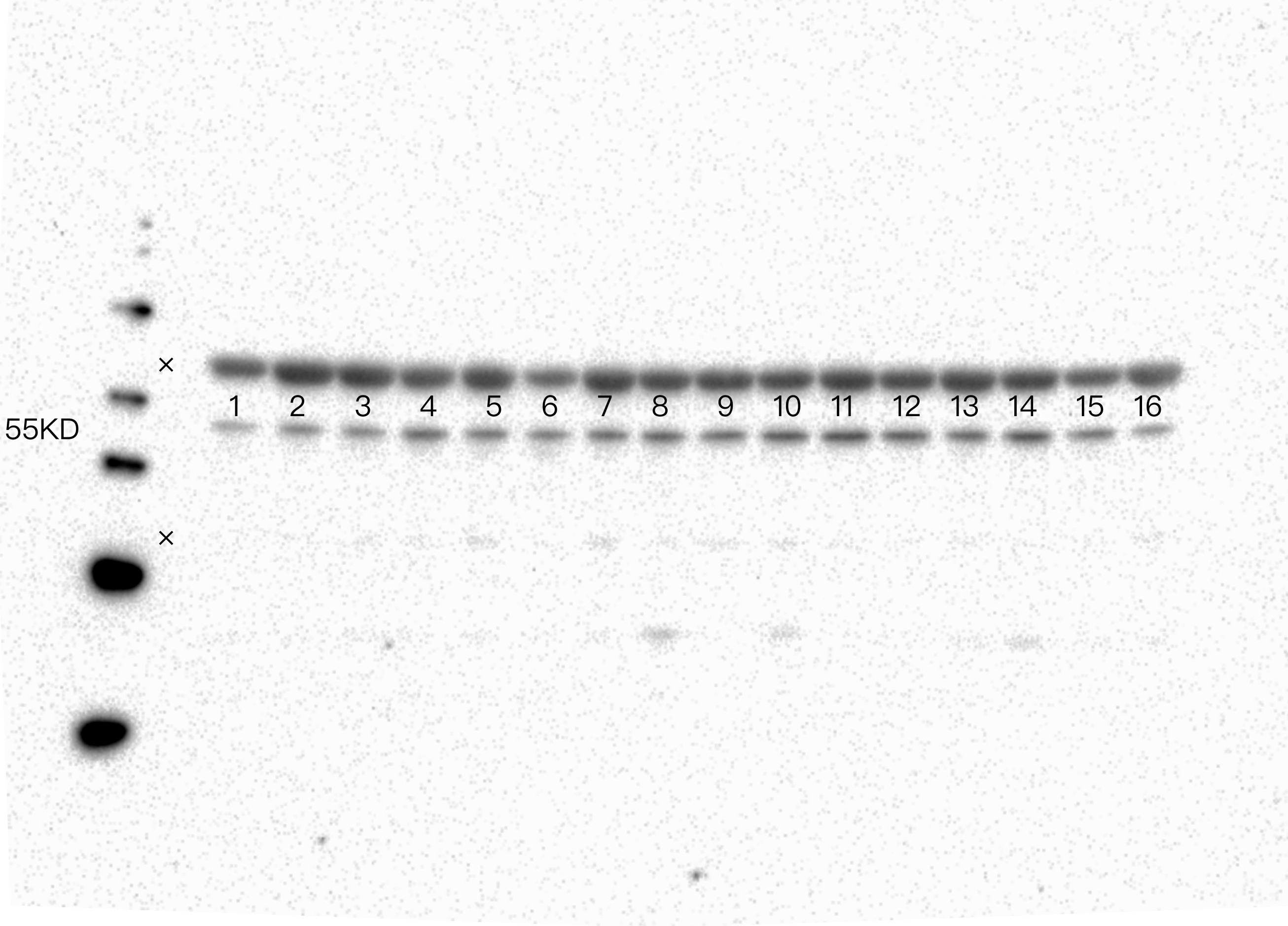

Sample ID
1. 2019070401
2. 2019070501
3. 2019070502
4. 2019032202
5. 2018120701
6. 2018120702
7. 2018121701
8. 2018122701
9. 2019070801
10. 2019070901
11. 2019071001
12. 2019050402
13. 2019081101
14. 2019082901
15. 2019081201
16. 2019080201

Antigen-antibody complexes were stained with Clarity Western ECL substrate(Bio-Rad) and visualization was performed using an image scanner(ChemiDoc XRS Plus Imaging System, Bio-Rad)

Tha samples were loaded in the order of their numbers.
In the figure 1-16 are shown.
Protein: P62

62KD

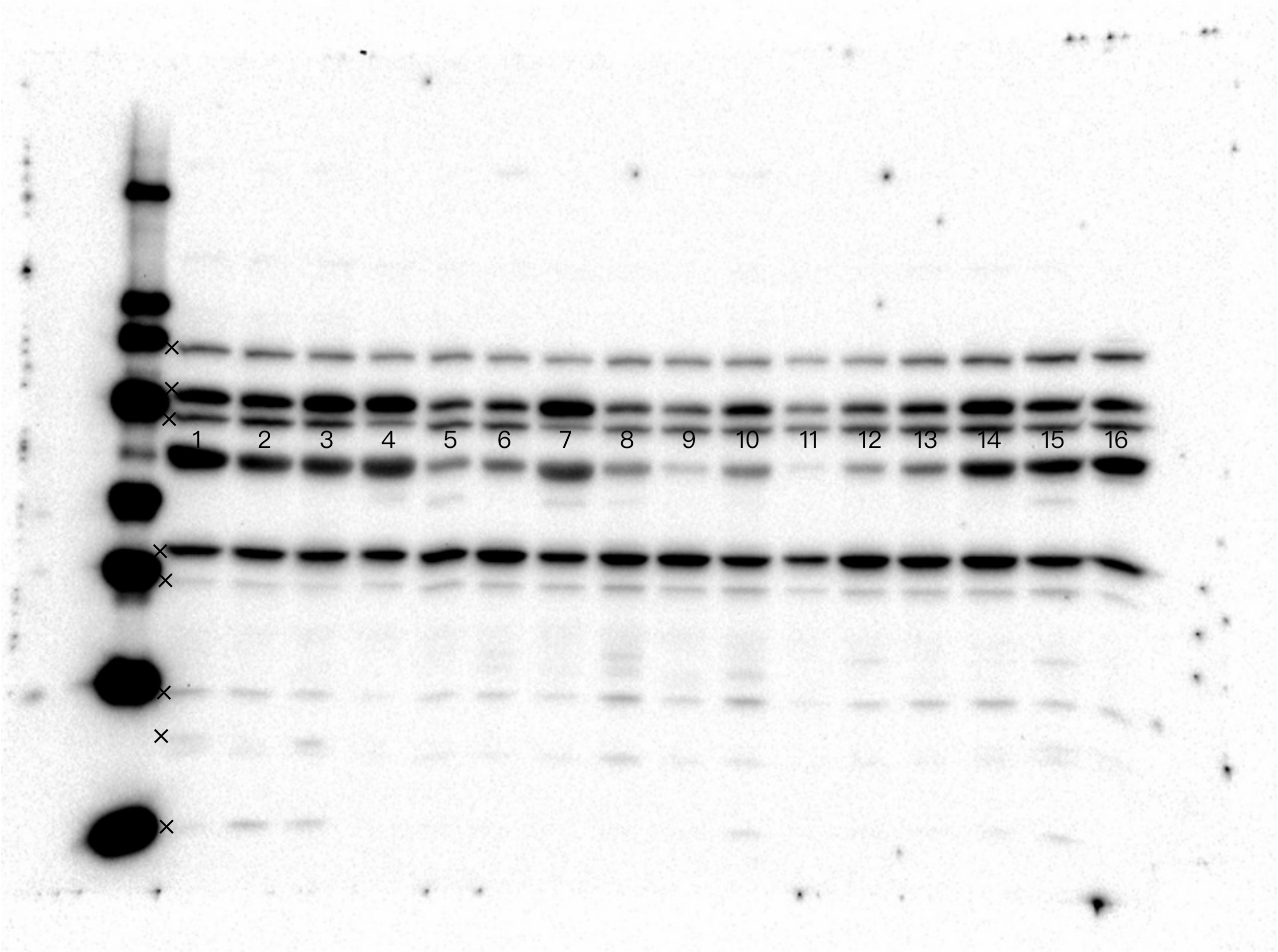

Sample ID
1 2019032202
2. 2019022202
3. 2019022302
4. 2019041301
5. 2018120702
6. 2018121901
7. 2018120702
8. 2019011001
9. 2019070801
10. 2019071001
11. 2019050402
12. 2019021301
13. 2019081201
14. 2019080201
15. 2019082001
16. 2019082801

Antigen-antibody complexes were stained with Clarity Western ECL substrate(Bio-Rad) and visualization was performed using an image scanner(ChemiDoc XRS Plus Imaging System, Bio-Rad)

Tha samples were loaded in the order of their numbers.
In the figure 1-16 are shown.
Protein: GAPDH

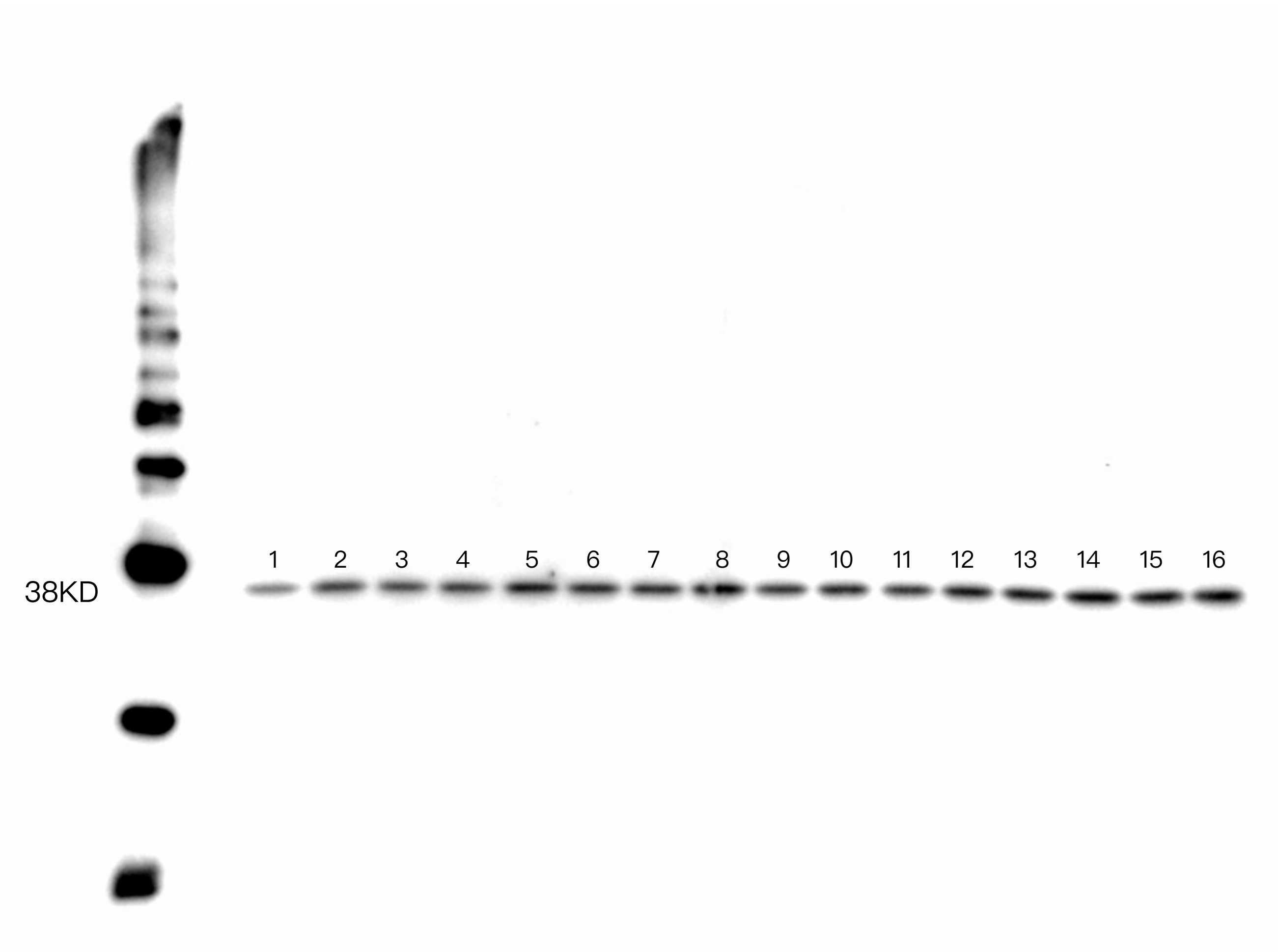

Sample ID
1. 2019070401
2. 2019070501
3. 2019070502
4. 2019032202
5. 2018120701
6. 2018120702
7. 2018121701
8. 2018122701
9. 2019070801
10. 2019070901
11. 2019071001
12. 2019050402
13. 2019081101
14. 2019082901
15. 2019081201
16. 2019080201

Antigen-antibody complexes were stained with Clarity Western ECL substrate(Bio-Rad) and visualization was performed using an image scanner(ChemiDoc XRS Plus Imaging System, Bio-Rad)
