# Supplementary material for: The protective effect of carbamazepine on acute lung injury induced by hemorrhagic shock and resuscitation in rats
Source: PLoS One. 2024 Oct 23;19(10):e0309622. doi: 10.1371/journal.pone.0309622 (PMC11498730; doi:10.1371/journal.pone.0309622)
Supplement: Attachment — Submitted filename: Response_to_Reviewers.docx [file pone.0309622.s006.docx]

Reviewer #1:

[1] The manuscript by Li et al., describes the protective effect of CBZ on ALI induced by HSR in rats. While the data show protective effect in all the parameters used in this study, the question remains whether CBZ can be protective if given after the initiation of HSR and at the time of resuscitation. the manuscript will be significantly strengthened by examining the effect of CBZ at the time of resuscitation.

Thank you for the insightful comments and the constructive critique of our manuscript detailing the effects of carbamazepine (CBZ) on acute lung injury (ALI) induced by hemorrhagic shock and resuscitation (HSR) in rats.

Your suggestion to investigate whether CBZ could be protective if administered during the resuscitation phase raises an important consideration for our study. We acknowledge the potential significance of this timing on the therapeutic efficacy of CBZ and appreciate the depth it could add to our findings.

In response to your comments, we have conducted additional experiments. We administered CBZ at the beginning of resuscitation and compared the pathological changes. We found that CBZ treatment at the time of resuscitation could also improve lung injury, although pretreatment with CBZ showed a better therapeutic effect.

We hope this amendment strengthens the manuscript and paves the way for future research in this direction. Thank you once again for your valuable feedback, which has undeniably enriched our study.

[2] In addition, details of the formulation of CBZ, concentration of DMSO used for the reconstitution etc are needed in the methods. It should also be clear in the methods that the drug was given prior to HS and resuscitation.

Thank you for the detailed review and insightful feedback on the methodology section of our manuscript.

We acknowledge the need for clarity and detail regarding the formulation of carbamazepine (CBZ) and the concentration of DMSO used for reconstitution. In response to your comments, we have revised the methods section to include detailed information about the preparation of CBZ, specifying the concentration of DMSO used and the steps taken to ensure proper dissolution and stability of the solution.

Additionally, we have made it explicitly clear in the methods section that CBZ was administered to the experimental subjects prior to the initiation of hemorrhagic shock and resuscitation (HS). This clarification ensures that the timing of drug administration is transparent and accurately reflects the experimental design intended to evaluate the protective effects of CBZ.

We appreciate your guidance in enhancing the precision and reproducibility of our experimental procedures. We hope these modifications address your concerns and improve the methodological rigor of our study.

Thank you once again for your valuable contributions to the refinement of our work.

[3] Lung MPO measurement is a good indicator for neutrophil infiltration in the lungs. I recommend the authors use either MPO in the lung tissues or Gr-1 histologically to assess neutrophil infiltration.

Thank you for the insightful comments and suggestions regarding our manuscript.

Regarding your recommendation to employ MPO or Gr-1 histological assessments for evaluating neutrophil infiltration in lung tissues, we recognize the value these additional experiments would add to our findings.

We checked MPO activity and compared it across different groups. Consistent with the neutrophil results, the CBZ treatment group showed a significant decrease in MPO activity compared to the HSR group.

Reviewer #2:

This seems like a well conducted and well written study. I have only minor comments:

[1] Line 60: most studies referenced in 12-17 are mouse studies, so it’s not clear that the word “patients” is appropriate here.

Thank you very much for your thoughtful and constructive comments regarding our manuscript. Your feedback has been invaluable to refining our study and strengthening the presentation of our findings.

Regarding your comment on Line 60, you rightly pointed out that the term "patients" may not be appropriate since the referenced studies primarily involve mouse models. After careful consideration, we have deleted this term to avoid confusion and more accurately reflect the nature of the studies involved.

[2] Line 62: The paper cited used both zebrafish and mice. Zebrafish don’t have lungs, so I think this sentence needs to be revised.

Thank you for your detailed review and insightful comments on our manuscript. We appreciate your guidance and the opportunity to improve our paper.

In response to your observation on Line 62, we have re-evaluated the section discussing the findings from the paper that utilized both zebrafish and mice. You correctly noted that zebrafish do not possess lungs, which is a critical anatomical distinction that impacts the interpretation of the results. We have revised this sentence to ensure its accuracy.

[3] Line 63: replace “another research” with “another study”?

Thank you for your detailed review and insightful comments on our manuscript. Regarding your suggestion on Line 63, we have replaced "another research" with "another study" to ensure the terminology is consistent and precise within the academic context. We agree that this change improves the formal tone and readability of our manuscript. We appreciate your guidance on these terminological adjustments and have carefully implemented them. We hope that these revisions meet your expectations and improve the overall quality of our paper.

[4] Lines 88,89 – please provide the dosage of chloroquine used.

Thank you for your meticulous review and attention to detail regarding our manuscript. In response to your comments on Lines 88 and 89, we have included the specific dosage of chloroquine used in our experiments. We have specified that the dosage administered was [10 mg/kg], which is consistent with the dosages commonly used in similar studies. This addition has been added to ensure the reproducibility of our results and to ensure full transparency regarding our experimental methods. We value your assistance in enhancing the accuracy and thoroughness of our study, and we hope that this addition meets the standards of the journal.

[5] Line 353: “level of P62 protein expression”?

Thank you for your careful attention to the details in our manuscript. In response to your comment on Line 353 concerning the "level of P62 protein expression," we have carefully reviewed our manuscript to ensure that the language accurately reflects the scientific measurements conducted.

We appreciate your guidance in enhancing the scientific rigor of our paper.
