# Supplementary material for: The protective effect of carbamazepine on acute lung injury induced by hemorrhagic shock and resuscitation in rats
Source: PLoS One. 2024 Oct 23;19(10):e0309622. doi: 10.1371/journal.pone.0309622 (PMC11498730; doi:10.1371/journal.pone.0309622)
Supplement: Attachment — Submitted filename: Response to Reviewers 20240807 .docx [file pone.0309622.s007.docx]

Dear Reviewers,

We would like to express our sincere gratitude to you for taking the time to review our manuscript. We appreciate your insightful comments and suggestions, which have greatly helped us improve the quality of our paper. Below, we have addressed each point raised by you.

**Reviewer #1:**

**Comment:** It is important to indicate in the discussion that the post treatment did not improve the outcome as similar to pre-treatment and consider this finding as a limitation of the study.

**Response:** Thank you for this important observation. We have revised the discussion section to clearly indicate that the post-treatment did not yield an improved outcome compared to the pre-treatment. We have also acknowledged this as a limitation of our study. The relevant changes can be found in lines 429–431.

**Reviewer #2:**

**Comment:** Overall, I think the manuscript has been improved, so I recommend accepting with minor revision. However, the text added to address the lack of experimental detail appears to have been copied from a protocol and needs grammatical revision. Additionally, there are a number of other places where I think the manuscript would benefit from edits in order to improve clarity. Some examples are below. I note that there are numerous online editing services that might be employed to improve the readability of the manuscript.

**Response:** We appreciate your positive feedback and constructive suggestions. We have carefully revised the manuscript to address the grammatical issues and improve clarity.

Specifically, we have sought the assistance of an online editing service to further improve the readability and overall quality of the manuscript.

Once again, we are grateful for your valuable feedback and the opportunity to improve our work. We hope that the revisions meet your expectations and that our manuscript is now suitable for publication.

Thank you for your time and consideration.

Best regards.
